# Original Antigenic Sin and Pandemic (H1N1) 2009

**DOI:** 10.3201/eid1606.091563

**Published:** 2010-06

**Authors:** Amesh A. Adalja, D.A. Henderson

**Affiliations:** University of Pittsburgh Medical Center, Baltimore, Maryland, USA

**Keywords:** Influenza, H1N1, pandemic (H1N1) 2009, viruses, vaccine, pandemic, letter

**To the Editor:** While pandemic (H1N1) 2009 was in its earliest stages, age distribution data indicated surprisingly few cases among persons >65 years of age. The initial assumption was that few persons >65 years of age had yet to be exposed. However, as more data became available from Mexico, Australia, and the United States, the age distribution pattern persisted (*1*).

This observation raised the question about whether older persons were protected from infection with an influenza virus A (H1N1) strain acquired many years ago. Indeed, data from the Centers for Disease Control and Prevention showed that approximately two thirds of older persons have evidence of immunity to pandemic (H1N1) 2009 virus. In 1960, Thomas Francis proposed the hypothesis of original antigenic sin, a phenomenon whereby a person who as a child was first exposed to a specific influenza virus A would, throughout life, mount an immune response to the virus of childhood, even when exposed to other antigenically dissimilar influenza viruses. In effect, the original antibody response generated by the immune system against a specific influenza viral strain was hypothesized to have colored all future responses to influenza (*2*).

Serologic responses of humans and other mammals have supported this theory. A new hemagglutinin (HA) subtype emerged in 1918 that was responsible for the pandemic that year. Through 1956, the strain evolved, accumulating mutations. In an era before influenza viruses were subtyped was performed, the original 1918 influenza virus A (H1N1) was dubbed a swine strain, whereas the virus of the 1930s was known as influenza A. However, the amount of drift accrued by 1947 was enough to render the seasonal vaccine of the time ineffective, and the new drifted virus strain was named A′. Throughout the period, the virus continued to be the subtype H1N1, as it is now designated.

In 1956, Davenport and Hennessy examined the antibody responses of 3 different age cohorts, each of which received different monovalent influenza vaccines prepared with vaccine strains circulating at different earlier periods (*3*) (Table). Prevaccination serum samples confirmed the presence of antibodies specific to the influenza virus that circulated during each respective cohort’s childhood.

**Table:** Influenza strains dominant for specific age cohorts from 1956 study*

Age cohort, y	Influenza strain
4–10 (born 1946–1952)	A′
17–28 (born 1928–1939)	A
>30 (born <1926)	Swine

*Adapted from (*3*).

Each of the 3 monovalent vaccines was administered to a group from each age cohort. Vaccination directed toward influenza strains distinct from the virus of childhood not only resulted in development of immunity to the vaccine strain but also boosted the immune response to the virus strain that circulated during each person’s childhood, i.e., original antigenic sin was apparent in each age cohort. Several other studies with humans, ferrets, rats, and rabbits yielded similar results (*4*,^5^).

Evidence from more recent studies largely supported the veracity of original antigenic sin. In a 1976 study, persons were vaccinated with a virus that circulated in 1973, an antigenically drifted variant of the 1968 influenza virus A (H3N2), and the response was assessed. As in earlier studies, examination of the antibodies generated indicated that the vaccine-induced antibodies were not only to the 1973 variant it contained but also to the virus that had circulated earlier. As the hypothesis postulates, the vaccine-induced antibodies to the 1968 strain were more numerous than those to the actual vaccine strain (*6*). Results from a 1984 experiment that used cell cultures with donor lymphocytes were similar (*7*). A 1994 study found that current vaccine strains induced antibodies to the influenza virus circulating during the childhood of persons in each age cohort (*8*). An additional study, published in 2009, confirmed the presence of antigenic sin in mice and showed a greater tendency for live-virus vaccines to produce the phenomenon (*9*).

One recent study is at variance with the others. It showed that monoclonal antibodies generated through vaccination were highly specific to the current vaccine strain rather than to influenza strains that had circulated in the past (*10*).

At the advent of the 2009 pandemic, fears of a severe pandemic were rampant. However, any prior immunity that was present in the population would dampen the impact of the virus. Early reports confirmed that the virus was less common in groups of older adults. Vaccine recommendations for certain age groups were developed according to that pattern of illness.

Because influenza virus A (H1N1) circulated continually after 1918 until 1957, most persons born before 1957 had been infected primarily with subtype H1N1. According to the theory of original antigenic sin, these persons may have partial protection from severe disease from infection with the new influenza virus A (H1N1), i.e., pandemic (H1N1) 2009. Supporting this hypothesis is the paucity of infections in Mexico from persons now in their 50s and 60s and few reports in the United States or Australia of cases in this age group (*1*). This fact should inform policy decisions and merits further immunologic consideration. Influenza surge planning is premised on a high incidence of illness among elderly persons, but if the current pattern of illness continues, healthcare facilities also should prepare to treat younger persons who may constitute the bulk of cases. Additionally, studies of persons born during 1957–1968 should be conducted to quantify antibody levels to pandemic (H1N1) 2009 virus, focusing on the degree of preexisting immunity that may have existed and was boosted by prior encounters with subtype H1N1 viruses
